# Loss of Bmp2 impairs odontogenesis via dysregulating pAkt/pErk/GCN5/Dlx3/Sp7

**DOI:** 10.21203/rs.3.rs-3299295/v1

**Published:** 2023-09-19

**Authors:** Shuo Chen, Feng Wang, Guobin Yang, Guohua Yuan, Mengmeng Liu, Graham Goldman, stephen harris, Wei Wang, Zhi Chen, MacDougall Mary

**Affiliations:** UT Health Science Center at San Antonio; Wuhan Univ; Wuhan University; School of Dentistry, the University of Texas Health Science Center at San Antonio; School of Dentistry, the University of Texas Health Science Center at San Antonio; U. of Texas Health Science Center at San Antonio; Fujian Medical University; Wuhan University School and Hospital of Stomatology; The University of British Columbia

**Keywords:** Bone morphogenetic protein 2, Dentinogenesis, Dentinogenesis imperfecta, Dentine sialophosphoprotein (DSPP), Dentine matrix protein 1 (DMP1), Transcriptional factors, histone acetyltransferases, Chromatin remodeling

## Abstract

BMP2 signaling plays a pivotal role in odontoblast differentiation and maturation during odontogenesis. Teeth lacking Bmp2 exhibit a morphology reminiscent of dentinogenesis imperfecta (DGI), associated with mutations in dentin matrix protein 1 (DMP1) and dentin sialophosphoprotein (DSPP) genes. Mechanisms by which BMP2 signaling influences expressions of DSPP and DMP1 and contributes to DGI remain elusive. To study the roles of BMP2 in dentin development, we generated Bmp2 conditional knockout (cKO) mice. Through a comprehensive approach involving RNA-seq, immunohistochemistry, promoter activity, ChIP, and Re-ChIP, we investigated downstream targets of Bmp2. Notably, the absence of Bmp2 in cKO mice led to dentin insufficiency akin to DGI. Disrupted Bmp2 signaling was linked to decreased expression of Dspp and Dmp1, as well as alterations in intracellular translocation of transcription factors Dlx3 and Sp7. Intriguingly, upregulation of Dlx3, Dmp1, Dspp, and Sp7, driven by BMP2, fostered differentiation of dental mesenchymal cells and biomineralization. Mechanistically, BMP2 induced phosphorylation of Dlx3, Sp7, and histone acetyltransferase GCN5 at Thr and Tyr residues, mediated by Akt and Erk^42/44^ kinases. This phosphorylation facilitated protein nuclear translocation, promoting interactions between Sp7 and Dlx3, as well as with GCN5 on Dspp and Dmp1 promoters. The synergy between Dlx3 and Sp7 bolstered transcription of Dspp and Dmp1. Notably, BMP2-driven GCN5 acetylated Sp7 and histone H3, while also recruiting RNA polymerase II to Dmp1 and Dspp chromatins, enhancing their transcriptions. Intriguingly, BMP2 suppressed the expression of histone deacetylases. we unveil hitherto uncharted involvement of BMP2 in dental cell differentiation and dentine development through pAkt/pErk42/44/Dlx3/Sp7/GCN5/Dspp/Dmp1.

## INTRODUCTION

Tooth development is the result of intricate interactions between epithelial-mesenchymal cells leading to matrix-producing cell differentiation. Odontoblasts originating from the neural crest, are responsible for synthesizing and secreting dentin extracellular matrix proteins, comprising both collagenous and non-collagenous proteins (NCPs) ^1,2^. Among NCPs, dentin sialophosphoprotein (DSPP) and dentin matrix1 (DMP1) exhibit high expression levels in odontoblasts and dentin. They play pivotal roles in odontoblast differentiation and mineralization processes ^3,4^. DSPP and DMP1 gene mutations in humans and mice caused dentinogenesis imperfecta (DGI) ^5–9^. The incidence of DGI in the population is estimated to be around 1 in 8,000 ^10^. Throughout odontogenesis, the differentiation of dental mesenchymal cells into odontoblasts is tightly regulated by an array of factors, including transcription factors and growth factors ^11^.

Belonging to the transforming growth factor-beta family, BMPs (bone morphogenetic proteins) were initially identified for their ability to induce ectopic bone formation ^12^. BMP2, a member of this family, exerts diverse biological functions ^12–14^. Within dental mesenchymal cells, BMP2 expression is prevalent ^15^. This protein plays a crucial role in driving the commitment of dental mesenchymal progenitor/stem cells ^16^ consequently promoting the odontoblast lineage. This process is achieved through the regulation of transcription factors using both the canonical and non-canonical BMP pathways ^12,14,17–20^. BMP2 significance extends to tooth development during embryonic and postnatal stages. Notably, mutations in BMP2 result in anomalies in tooth morphology, mirroring the symptoms observed in DGI ^2,6,8,21,22^. Nonetheless, the precise mechanisms by which BMP2 operates during dentinogenesis remain to be fully elucidated.

Transcription factors DLX3 and Sp7 (also known as Osterix, Osx) play indispensable roles in the differentiation of both odontoblasts and osteoblasts. They are integral components of the signaling pathways downstream of BMP2, contributing to osteogenesis and odontogenesis ^22–25^. These factors, DLX3 and Sp7, are expressed within both dental mesenchyme and epithelium ^14,22^. They exert their effects by initiating lineages of osteoblasts and odontoblasts through various signaling pathways ^14,26^. Notably, mutations in DLX3 and Sp7 have been linked to bone and tooth defects in both human and mouse studies ^23,27–30^.

GCN5 is one of the histone acetyltransferases (HATs) ^31^. Its actions encompass acetylating histones as well as non-histone proteins, which include transcription factors ^32^. Operating as a coactivator, GCN5 contributes to transcriptional activity by facilitating the unfolding of chromatin, allowing for nucleosome assembly and transcription to occur ^33,34^. The importance of GCN5 extends to various biological processes such as embryonic development and cell differentiation ^35,36^. GCN5 gene KO mice cause early embryonic lethality and increased apoptosis in mesodermal cell lineages ^37^. Nevertheless, the mechanisms governing GCN5 involvement in odontoblast differentiation remain largely unexplored, and the role of Bmp2 in this context via GCN5 signaling remains elusive.

In this study, we present a novel Bmp2 signaling pathway implicated in odontogenesis. Our findings demonstrate the indispensability of Bmp2 in driving odontoblast differentiation through the regulation of genes associated with tooth development. Furthermore, Bmp2 mediates the phosphorylation of Dlx3, Sp7, and GCN5 at tyrosine (Tyr) and threonine (Thr) residues via Akt/Erk^42/44^ kinases, thereby facilitating their translocation into the nucleus. This translocation, in turn, enables crucial protein-protein interactions that modulate the chromatins of Dspp and Dmp1. Notably, phosphorylated GCN5 translocates to the nucleus, leading to the acetylation of Sp7 and histone H3 at lysine (Lys) residues. This acetylation is instrumental in recruiting RNA Pol II to the chromatins of Dspp and Dmp1, ultimately activating their transcription processes.

## RESULTS

### Abnormal tooth structure in Bmp2 cKO mice

**Figure 1:**
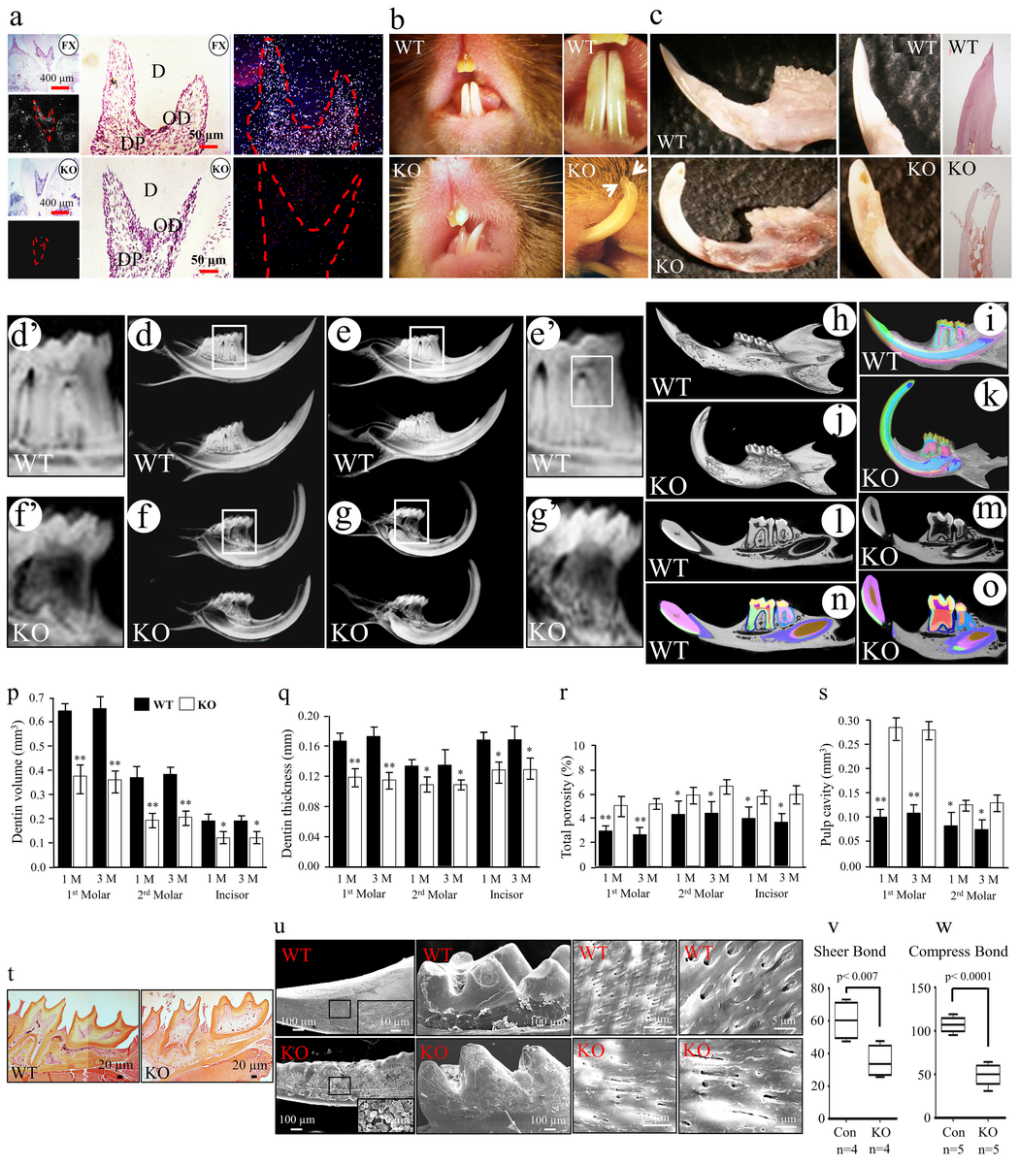
Abnormal tooth structure in Bmp2 knockout mice. (a) In situ hybridization assay. Anti-sense mRNA of Bmp2 exon 3 gene as the probe was used to detect Bmp2 mRNA expression within odontoblasts and dental pulp cells at the 21-day old Bmp2fx/fx mice (bright signal) and at the same age of the Bmp2 cKO mice. FX, floxed; KO, Bmp2 knockout; D, dentin; Dp, dental pulp chamber; Od, odontoblasts. (b) In the Bmp2 mutant mice, both sides of the up and lower incisors were asymmetric with open forked. The incisor edge was jagged with chipping and the tip incisor wearing (arrows). (c) Morphology of teeth revealed abnormal incisors and incisor dental pulp chambers in the Bmp2 cKO teeth were exposed. (d-g’) X-ray analysis of the teeth in the control (d, e) and Bmp2-cKO (f, g) mice. The mineral density of incisors and molars from the Bmp2-cKO mice was decreased compared to the control mice. Abnormal morphology of the molars and incisors in the Bmp2 null mice was observed. The dental pulp cavity of the incisors from the 3-month-old null mice was exposed and the dental pulp cavity of the molars was enlarged, and thin dentin layer compared to that of the control-type teeth. d’, e’, f’, and g’ show higher magnification from the boxes of d, e, f, and g. (h-o) Micro-CT analysis of teeth of the control (h, i, l, n) and Bmp2 cKO (j, k, m, o) mice from 3-month-old mice. (p, q, r, s) Dentin volume, thickness, total porosity, and dental pulp chamber from the first, and second mandibular molars and incisors in the 1- and 3-month-old wild type and Bmp2 null mice were analyzed by Micro -CT. Bars show mean ± S.E. from three animals of each group. t-test, *p<0.05; **p<0.01. (t) The dentin layer of the mandibular molars, root development, and size of dental pulp cavity from 1-month-old wild type and Bmp2 cKO mice were observed by histological staining. (u) SEM of the mandibular incisors and molars from Bmp2 null and control mice. The enamel layer of the incisor and molar is rough as well as incisor surface and molar cusps are rugged and abraded in 3-month-old Bmp2 cKO mice whereas the enamel surface of the incisor and molar of the same age of the control mouse is smooth. The dentinal tubules and inter-tubular dentin are asymmetrically distributed, and the dentinal tube numbers are less and small in size in the Bmp2 mutant mice while the dental tubules and inter-tubular dentin in the control mice are uniform. (v, w) Tooth hardiness and elasticity between the control and Bmp2 cKO mice from 1-month-old mice were conducted by an electromechanical testing machine to measure shear and compressive strength. There are statistically significant differences by t-test (p<0.007, p<0.0001).

To investigate the role of Bmp2 in odontogenesis, Bmp2 conditional knockout (cKO) mice were generated (Fig. S1a-d). In situ hybridization confirmed minimal expression of the Bmp2 gene in dental pulp cells and odontoblasts of the mutant mice (Figs. 1a and Fig. S2). The Bmp2 cKO mice exhibited chalky white mandibular incisors with attrition and exposed dental pulp cavities (Fig. 1b, c). X-ray and Micro-CT analyses revealed abnormalities in mandibular incisors and molars, accompanied by a reduction in dentin mineral density in the mutants. Additionally, the null mice displayed thin dentin layers, wide dental pulp chambers, and delayed root development (Fig. 1d–o). The Bmp2 null mice displayed reduced dentin volume and thin dentin layers in mandibular incisors and molars. Enlarged dental pulp chambers with varying porosity were also evident (Fig. 1p–s). Histological examination supported these findings (Fig. 1t). SEM analysis indicated rough incisor and molar surfaces, sparse distribution of fissures and tubular dentine, and scattered dentinal tubules in the Bmp2 mutant mice (Fig. 1u). The dentin structure in Bmp2 cKO mice resembled that seen in cases of DGI ^6,9^. Mechanical testing revealed that the shear and compressive characteristics of the mutant mouse incisors were 1.72- and 2.15-fold weaker than those of the controls (Fig. 1v, w), indicating increased brittleness and reduced hardness and elasticity in the mutant incisors.

### Bmp2 signaling in dentin development

**Figure 2:**
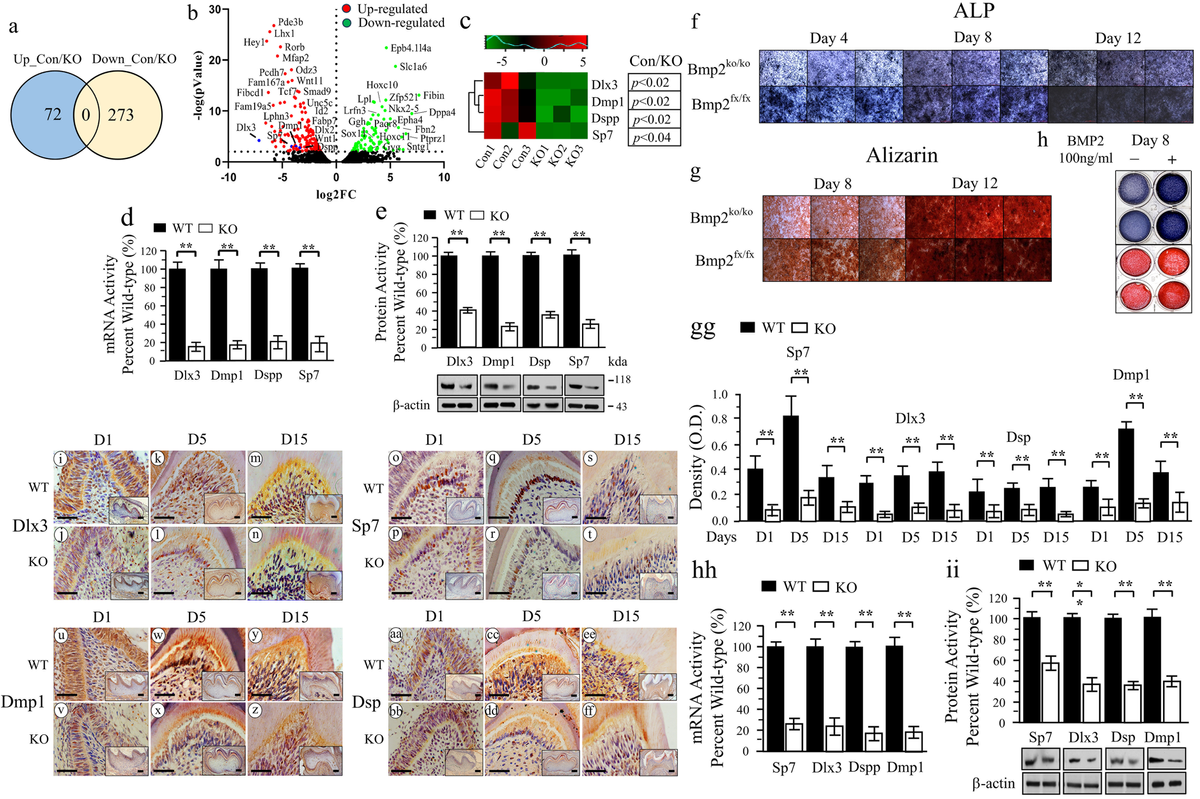
RNA-seq and gene expression. (a) RNA-seq. Total RNAs were isolated from the Bmp2ko/ko and Bmp2fx/fx dental mesenchymal cells and differentially expressed genes (DEGs) were selected by DESeq based on BHadjPvaland fold changes using RNA-seq. The ratio of Bmp2fx/fx /Bmp2 ko/ko dental mesenchymal cells was 72 upregulated and 273 downregulated. (b) Volcano plots of RNA-seq data, showing the genes up- or downregulated in Bmp2fx/fx vs Bmp2ko/ko dental mesenchymal cells. Dots in black are those genes that did not meet the criteria of being significantly expressed with a twofold change or greater. Thresholds appear as black dashed lines on the y-axis for significance (p Value < 0.01), y-intercept at −Log2FC = 2, and on the x-axis for foldchange (FC). Green dots indicated the downregulated genes. Red dots indicated the upregulated genes. n =3. (c) Heatmap comparison of gene expression profiles from the iBmp2fx/fx vs Bmp2ko/ko dental mesenchymal cells showed expression of Dlx3, Dmp1, Dspp, and Sp7 was decreased. (d) Expression of Dlx3, Dmp1, Dspp, and Sp7 in the Bmp2fx/fx vs Bmp2ko/ko dental mesenchymal cells was tested by RT-qPCR. The bar graphs show mean ± S.D (n = 3). t-test, **p<0.01. (e) Protein expression of Dlx3, Dmp1, Dsp, and Sp7 was detected by Western blotting. Expression of these proteins in Bmp2fx/fx dental mesenchymal cells acts as 100%. Bars show mean ± S.D. (n = 3) from three independent experiments: t-test, *p<0.05, **p<0.01. (f, and g) Cell differentiation and biomineralization in Bmp2fx/fx and Bmp2ko/ko dental mesenchymal cells were detected by ALP assay and Alizarin red S dye. These cells at days 8 and 12 in the calcifying medium were stained with Alizarin red S dye. (h) Cell differentiation and mineralization activities from Bmp2ko/ko dental mesenchymal cells treated with or without BMP2 (10 ng/ml) were measured by ALP and Alizarin red S assays. (i-ff) Immunohistochemistry showed expressions of Dlx3, Dmp1, Sp7, and Dsp within odontoblasts in control and Bmp2 cKO mice at postnatal days, 1 (D1), 5 (D5) and 15 (D15). Inserted photos represented lower magnification of the figures. Bars, 200 μm. (gg) The immunostaining density of Sp7, Dlx3, Dsp, and Dmp1 proteins within odontoblasts in the control and Bmp2 cKO mice was analyzed by J image software. Bars represent the mean ± S.E. (hh) RT-qPCR analysis of mRNA expression of Dlx3, Sp7, Dspp, and Dmp1 from control and Bmp2 cKO mice at D1. Values of control mRNAs were expressed as a 100-fold increase versus Bmp2 cKO mice. (ii). The expressions of Dlx3, Sp7, Dsp, and Dmp1 for the control and Bmp2 cKO mice at D1 were analyzed by Western blotting. Up panel showed the quantified data assessed by ImageJ software. Bars represent the mean ± S.E. from three animals in three separate experiments. t-test, *p < 0.05; **p < 0.01.

To study the mechanisms underlying Bmp2 influence on odontogenesis, RNA-seq was employed to evaluate gene expression in dental mesenchymal cells from Bmp2^fx/fx^ and Bmp2^ko/ko^ mice. This analysis identified 22,609 genes that displayed differential expression, with 273 genes downregulated and 72 upregulated in Bmp2^fx/fx^ dental mesenchymal cells (Fig. 2a, b). Notably, this set of differentially expressed genes included Dlx3, Dmp1, Dspp, and Sp7, all of which have related to odontogenesis (Figs. 2c). These findings were further corroborated using RT-qPCR and Western blot analyses (Fig. 2d, e). In vitro assessments of dental mesenchymal cells lacking Bmp2 (Bmp2^ko/ko^) showed impairments in cell differentiation and mineralization (Fig. 2f, g). Intriguingly, cell differentiation and mineralization in Bmp2^ko/ko^ dental mesenchymal cells were rescued by BMP2 (Fig. 2h). Subsequent in vivo studies illuminated decreased expression of Dlx3, Dmp1, Dspp, and Sp7 within odontoblasts in Bmp2 cKO mice (Fig. 2i–ii), aligning with findings from the in vitro investigations (Fig. 2b–e).

### BMP2 facilitates phosphorylation of Dlx3, Sp7, and GCN5 via Akt and Erk^42/44^ kinases

**Figure 3:**
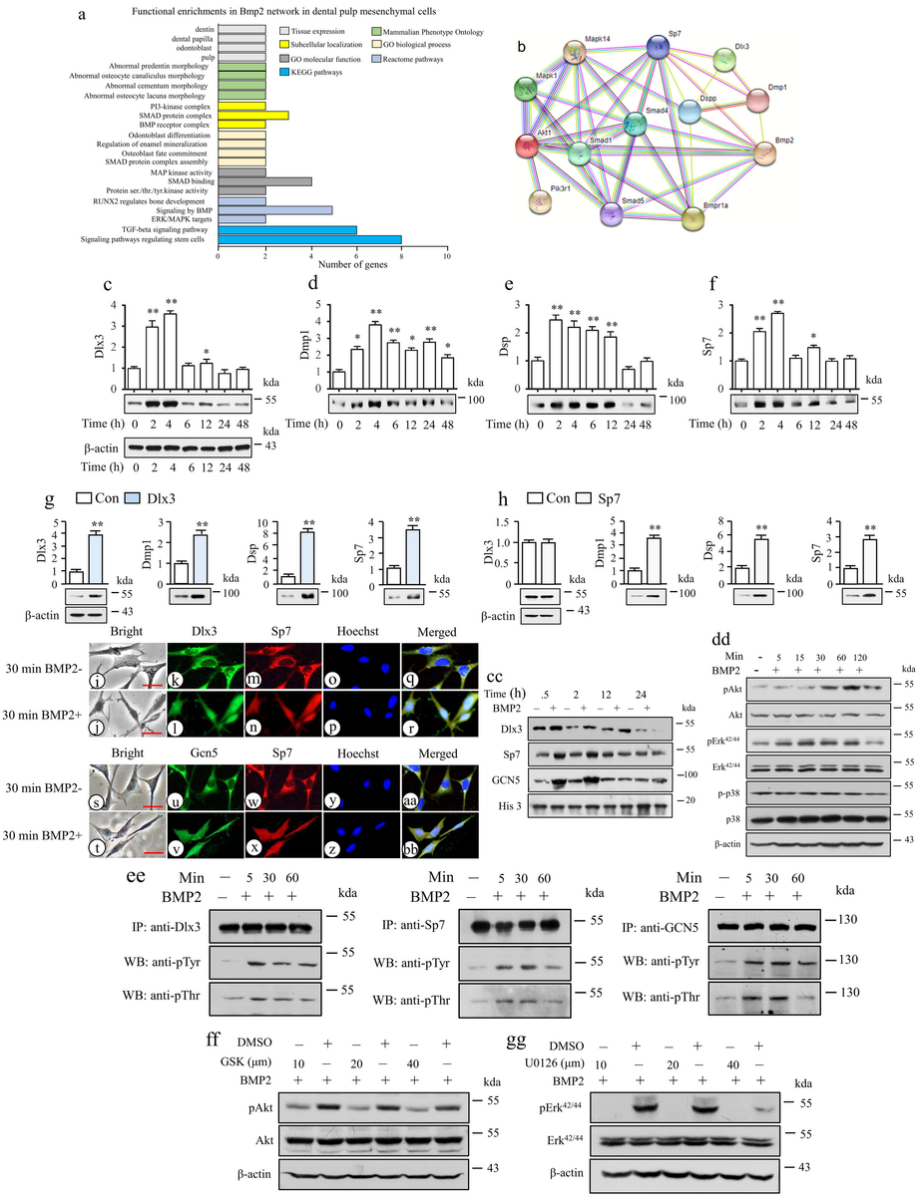
Effect of BMP2 on expression of Dlx3, Sp7, Dspp and Dmp1 in mouse dental mesenchymal cells. (a) KEGG and GO represent BMP2 signal pathways involved in functional enrichments of dentinogenesis and others. (b) PPI predicts the network interaction of BMP2 with its target genes including canonical and non-canonical BMP signal pathways as well as Dlx3, Dmp1, Dspp, and Sp7. (c-f) The cells were treated with or without recombinant BMP2 (100 ng/ml) for 0–48h and proteins were isolated and quantitated. The expression of Dlx3, Dmp1, Dsp, and Sp7 was detected by Western blot, and β-actin was used as an internal control. These protein expressions in 0 h served as a 1.0-fold increase. Bars represent the mean ± S.E. from three separate experiments. t-test, *p < 0.05; **p < 0.01, indicates significant differences. (g, h) The cells were transfected with pcDNA-Dlx3 or pcDNA-3.1 (g) and with pcDNA-Sp7 or pcDNA 3.1 (h) for 48h. After transfection, protein expression of Dlx3, Dmp1, Dsp, Sp7, and β-actin was detected by Western blotting. The fold activity was determined by individual value by the control group value (pcDNA 3.1). Up panels were quantitated by Image software and bars represent the mean ± S.E. from three separate experiments. t-test, *p <0.05; **p <0.01. I-BB. Effect of BMP2 on nuclear translocation of Dlx3, Sp7, and GCN5 in iMDP3 cells. The cells were treated with or without 100 ng/ml of recombinant BMP2. Co-expression of Dlx3 (green, k, l), Sp7 (red, m, n), GCN5 (green, u, v), and Sp7 (red, w, x) was observed in the cells by fluorescent images. (i, j, s, t) bright images, Hoechst for nuclei (o, p, y, z), merged images (q, r, aa, bb). Bars, 10 μm. (cc) Cells were treated or untreated with BMP2 in 0.5h, 2h, 12h, and 24h, and nuclear extracts were then isolated and quantitated. Expression of Dlx3, Sp7, GCN5 and histone H3 (His 3) proteins in nuclear extracts was detected by Western blotting. (dd) Cells were treated or untreated with BMP2 in the given periods, and proteins were isolated and quantitated. Expression of pAkt, Akt, pErk42/44, Erk42/44, p-p38, p38, and β-actin as control was detected by Western blotting. (ee) Cells were treated with BMP2 for 0–60min, and Dlx3, Sp7, and GCN5 proteins were immunoprecipitated using anti-Dlx3 or anti-Sp7, anti-GCN5 antibodies, respectively. Phosphorylation of Dlx3, Sp7, and GCN5 at Tyr and Thr residues was assayed using anti-pTyr or anti-pThr antibodies, respectively. (ff, gg) Cells were treated or untreated with 10 μM of U0126 or GSK630639 (GSK), respectively for 2h and followed by adding BMP2. Nuclear extracts were then isolated and quantitated. Expression of pAkt, Akt, pErkl42/44, Erk42/44, and β-actin as control from the nuclear extracts were analyzed by Western blotting.

Gene Ontology (GO) revealed that BMP2 significantly enriches dental mesenchymal cell differentiation and odontogenesis, operating through several signaling pathways including TGF-β, MAPK, and PI3K-AKT (Figs. 3a and S3a-c). Network analysis underscored Bmp2 regulatory impact on Dlx3, Dmp1, Dspp, and Sp7 via both canonical and non-canonical BMP pathways (Fig. 3b). In mouse dental mesenchymal cells (iMDP3), BMP2 stimulation notably prompted the expression of Dlx3, Dmp1, Dspp, and Sp7 (Fig. 3c–f). Given the transcription factor roles of Dlx3 and Sp7, we probed whether BMP2 influence on Dmp1 and Dspp expressions is mediated through Dlx3 and Sp7. Elevation of Dlx3 or Sp7 gene levels in iMDP3 cells yielded an increase in Dmp1 and Dspp expressions (Fig. 3g, h). Notably, Dlx3 boosted Sp7 expression, whereas Sp7 exerted no influence on Dlx3. This indicated that Sp7 functions as a downstream target of Dlx3. Further evidence showed that specific Dlx3-siRNAs reduced expression of Dlx3, Dmp1, Dspp, and Sp7 (Fig. S4a). However, Sp7-siRNA solely diminished Sp7, Dmp1, and Dspp expressions, with no effect on Dlx3 (Fig. S4b). TESS analysis identified four Dlx3 binding sites in the mouse Sp7 promoter, and ChIP assays confirmed Dlx3 interaction with these motifs (Fig. S4c-d). These findings collectively indicate that BMP2 induces Dmp1 and Dspp transcription via Dlx3 and/or Sp7 signaling.

Biological roles for Dlx3 and Sp7 manifest predominantly within cell nuclei. Dlx3 and Sp7 proteins were primarily located in the cytoplasm and weakly in nuclei without BMP2 treatment. BMP2 induction exhibited a shift with their distribution mainly occurring within nuclei (Figs. 3i–bb and S5). Western blotting supported this finding (Fig. 3cc). Moreover, BMP2 also spurred the translocation of GCN5 and Sp7 proteins from the cytoplasm to the nucleus (Figs. 3u–x and S5). Intriguingly, both Dlx3 and Sp7 were co-expressed in nuclei (Fig. 3k–bb). This dynamic interplay underscores how BMP2 orchestrates the intracellular distributions of Dlx3, Sp7, and GCN5.

To decipher the mechanism driving BMP2 induction of nuclear translocation for Dlx3, Sp7, and GCN5, we found that BMP2 not only instigates phosphorylation of Akt and Erk^42/44^ (Fig. 3dd), but also of Dlx3, GCN5, and Sp7 at Tyr and Thr residues (Fig. 3ee). Interestingly, the use of Akt (GSK690693) or Erk (UO126) inhibitors impeded the phosphorylation of Dlx3, GCN5, and Sp7 mediated by Akt and Erk^42/44^ kinases in response to BMP2 (Figs. 3ff, gg, and S6a-h). These findings collectively affirm that BMP2 activates the phosphorylation and nuclear translocation of Dlx3, Sp7, and GCN5 through the Akt and Erk^42/44^ pathways mediated by BMP signaling.

### BMP2 upregulates Dmp1 and Dspp transcriptions through Dlx3 and Sp7.

**Figure 4:**
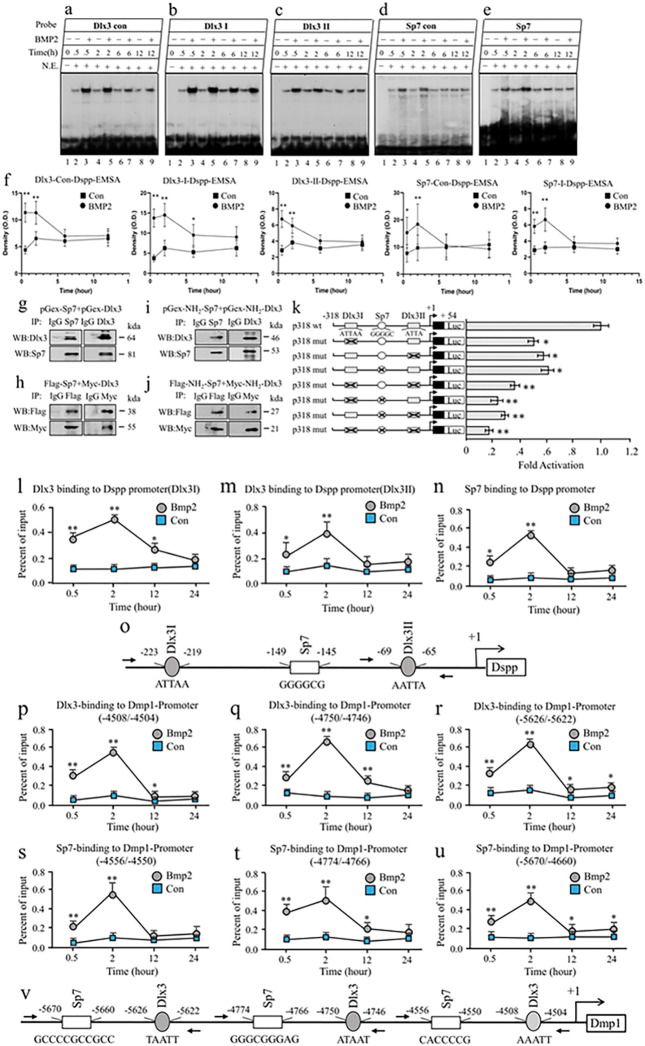
BMP2 upregulation of Dmp1 and Dspp transcriptions via Dlx3 and Sp7. (a-e) The binding affinity of Dlx3 and Sp7 to their binding sites is induced by BMP2. The cells were treated with or without BMP2 (100 ng/ml) for 0, 0.5, 2h, 6h, and 12h. Nuclear extracts were isolated and quantitated. The binding activity of Dlx3 and Sp7 in their motifs in the Dspp promoter was observed by EMSAs. (f) Dynamic effect of BMP2 on the binding of Dlx3 and Sp7 to Dspp promoter detected by EMSA. The cells were treated with or without 100 ng/ml of BMP2 at 30 min, 2h, 12h, and 24h. Then, nuclear extracts were isolated and the binding of Dlx3 and Sp7 to their motifs in the Dspp promoter was analyzed by EMSA. Three independent experiments were performed. p*<0.05 and p**<0.01, shows significant differences. (g-j) For in vitro assay, the different fragments of Dlx3 and Sp7 genes were subcloned into pGex vectors, respectively, and the fused proteins were expressed and purified. In vitro interaction assay was carried out by incubating purified GST full-length Sp7 with GST full-length Dlx3 (g), GST NH_2_-Sp7, and GST NH_2_-Dlx3. (h) Immunoprecipitation was performed using anti-Sp7 or anti-Dlx3 antibodies. IgG was the negative control. Protein-protein interaction was detected by immunoblotting. For in vivo study, Co-IP assay was performed using whole cell extracts from iMDP3 and HEK293T cells expressing Flag-tagged full-length Sp7, and Myc-tagged full-length Dlx3 (i), Flag-tagged NH_2_-Sp7 and Myc-tagged NH2-Dlx3 (j). Anti-Flag, anti-Myc antibodies, and negative control IgG were pulled down by Co-IP. Anti-Flag or anti-Myc antibody was used for Western blotting to confirm the interaction of Dlx3 and Sp7. (k) Schematic representation of wild-type and mutant constructs of Dlx3 and Sp7 binding sites in the mouse Dspp promoter. Cells were transfected with wild-type or mutant chimeric or pGl3-basic and pRT-TK plasmids. The value (ratio firefly/Renilla Luc) was obtained, and the Luc activity of the wild-type group acts as a 1-fold increase. The fold Luc activity was calculated by dividing the individual value by the wild-type group value. The data show the mean ± S.E. from three separate experiments performed in triplicate. There are significantly different if *p<0.05, **p<0.01 by t-test. Mutant Dlx3 and Sp7 sites are shown by rectangles and round with cross lines. (l-o, and p-v) The dynamic effect of BMP2 on Dmp1 and Dspp transcriptions via Dlx3 and Sp7. The cells were treated with or without 100 ng/ml of BMP2 at 0.5h, 2h, 12h, and 24h. ChIP assay was used to pull down the binding sites of Dlx3 and Sp7 in the Dspp and Dmp1 promoters using anti-Dlx3 or anti-Sp7 antibodies. The dynamic binding affinity of Dlx3 (l, m), Sp7 (n) to their binding motifs in the Dspp promoter and of Dlx3 (p-r), and Sp7 (s-u) in the Dmp1 promoter was measured by qPCR using the specific primers. (o, and v) Schematic representation of Dlx3 and Sp7 binding sites in the Dspp (o) and Dmp1 (v) promoters. +1 represents transcriptional start sites. Arrows show primer positions designed. Mean data ± S.E. from three independent q-PCR experiments in triplicate was plotted. t-test, *p<0.05 and **p<0.01, show significant differences.

DSPP serves as a critical marker in the context of dentinogenesis ^6^. To elucidate the mechanisms by which BMP2 influences Dspp expression via Dlx3 and Sp7, reporter constructs of various Dspp promoters were combined with Dlx3 or Sp7 expression vectors and introduced into iMDP3 cells. This investigation identified pivotal regulatory regions, with the primary elements for Dlx3 and Sp7 located at nt −318 and + 54 (Fig. S7a, b). Further analysis through Vista plots revealed high conservation in DNA sequences within these regions across mouse, rat, and human genomes (Fig. S7c). Employing TESS software, we identified two Dlx3 and one Sp7 binding sites within the Dspp promoter, situated between nt −318 and + 54. Notably, the DNA sequences within these elements exhibited a high degree of homology across human, rat, and mouse genomes (Fig. S7d). Utilizing EMSA assays, we confirmed that Dlx3 and Sp7 probes formed complexes with nuclear extracts, indicative of binding (Fig. S7e, f). Furthermore, anti-Dlx3 and anti-Sp7 antibodies facilitated the formation of DNA-protein-antibody complexes, affirming their binding to Dlx3 and Sp7 proteins (Fig. S7e, f). Overexpression of the Dlx3 or Sp7 genes substantially intensified the interaction of Dlx3 and/or Sp7 proteins with their respective sites in the Dspp promoter, as observed via ChIP assays (Fig. S7g). In addition to BMP2 influence on the nuclear translocation of Dlx3 and Sp7 (Fig. 3r, bb), the binding affinity of these proteins to their sites within the Dspp regulatory element was augmented by BMP2 induction in iMDP3 cells, as demonstrated by EMSAs (Fig. 4a–f).

Given the co-expression of Dlx3 and Sp7 within dental cells (Fig. 3r, bb), investigations were conducted to ascertain whether these proteins exhibit mutual interactions. Both in vitro and in vivo assays using Co-IP revealed interactions between the NH_2_-termini of Dlx3 and Sp7 proteins (Fig. 4g–j). Further experimentation through transfection reporter assays revealed that concurrent mutation of Dlx3 and Sp7 binding sites led to a substantial reduction in promoter activity, a phenomenon not observed when only one of these sites was mutated. This points to a synergistic effect of Dlx3 and Sp7 on Dspp transcription (Fig. 4k).

To examine the dynamic effect of BMP2 on Dspp transcription via Dlx3 and Sp7, iMDP3 cells were subjected to BMP2 treatment over distinct time periods. As illustrated in Fig. 4l–o, the binding activity of Dlx3 and Sp7 to their respective motifs within the Dspp promoter experienced significant enhancement due to BMP2 treatment. The same trend was observed in the context of BMP2 influence on Dlx3 and Sp7 binding to the Dmp1 promoter (Fig. 4p–v). Collectively, these findings provide evidence that BMP2 governs the expression of Dmp1 and Dspp genes via Dlx3 and Sp7 in dental mesenchymal cells.

### GCN5 acetylates Sp7 and regulates Dmp1 and Dspp transcriptions by BMP2-GCN5-RNA polymerase II

**Figure 5:**
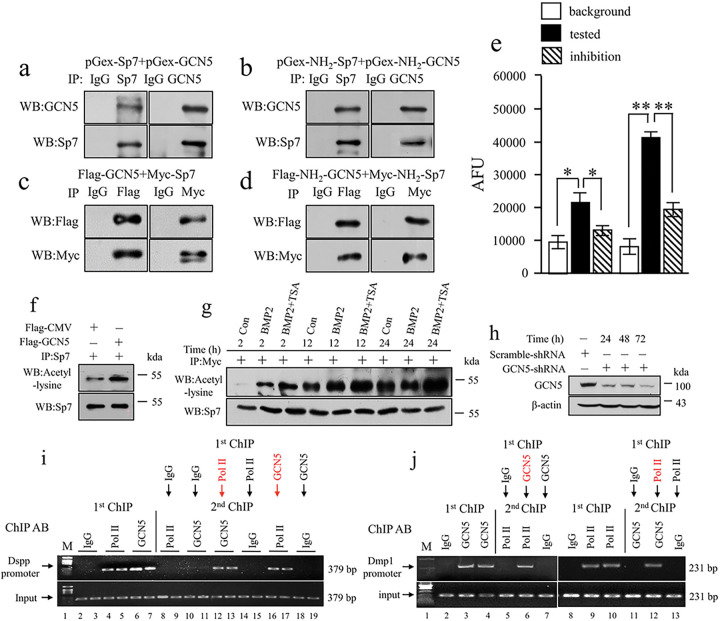
GCN5 acetylates Sp7 and regulates Dmp1 and Dspp transcriptions by BMP2-GCN5-RNA polymerase II. (a-d) For in vitro assay, the different fragments of Sp7 and GCN5 genes were subcloned into pGex vectors, respectively and fused proteins were expressed and purified. In vitro interaction assay was performed by incubating purified GST full-length Sp7 with GST full-length GCN5 (a), GST NH2-Sp7, and GST NH2-GCN5 (b). Immunoprecipitation was carried out by anti-Sp7 or anti-GCN5 antibodies. Protein-protein interaction was detected by Western blotting. IgG was as control. For in vivo study, Co-IP assay was performed using whole cell extracts from iMDP3 and HEK293T cells expressing Flag-tagged full-length Sp7 and Myc-tagged full-length GCN5 (c), Flag-tagged NH2-Sp7 and Myc-tagged NH2-Dlx3 (d). Sp7 and GCN5 proteins were used to pull down by Co-IP using anti-Flag or Myc antibody. Western blotting was subject to confirm the interaction between Sp7 or GCN5. (e) p300 and GCN5 acetylated Sp7, however, p300 and GCN5 activity were inhibited by anacardic acid. p300 and GCN5 (50 ng) activity assays were carried out with the fluorescent HAT assay using either GCN5 (50 ng) or p300 (50 ng), or Sp7 protein (50 μM), acetyl-CoA (50 μM) and anacardic acid (15 μM) for inhibition. The fluorescent signal was monitored on a SpectraMAX Gemini XS plate reader with excitation at 365 nm and emission at 470 nm. AFU, arbitrary fluorescence units. (f) Overexpression of GCN5 stimulated Sp7 acetylation in vivo. The cells were transfected with pcDNA GCN5 or pcDNA 3.1 plasmids for 48h, respectively. After transfection, the cells were lysed and immunoprecipitated by the anti-Sp7 antibody. The immunoprecipitation complex was blotted with anti-Sp7 and anti-acetyl-lysine antibodies. (g) Cells were transfected with pcDNA-Myc-Sp7. After 12h transfection, the cells were treated or untreated with 10 nM of TSA alone or plus 100 ng/ml of recombinant BMP2 for 2h, 12h, and 24h, respectively. Sp7 was pulled down by immunoprecipitation using an anti-Myc antibody. Sp7 acetylated lysine site (s) and Sp7 were detected by Western blotting using anti-acetyl-lysine or anti-Sp7 antibodies, respectively. (h) The cells were transfected with either scramble-shRNA or shRNA-GCN5 plasmid for 24h, 48h, and 72h. The expression of GCN5 and β-actin was detected by Western blotting using anti-GCN5 and anti-β actin antibodies. (i, j) Cells were subjected to the first ChIP assay of the Dspp and Dmp1 promoters using anti-GCN5 or anti-RNA pol II antibody or non-immune IgG as a negative control. For the Re-ChIP assay, the beads from the first ChIP with anti-GCN5 antibody were washed, eluted, and subjected to the second ChIP with anti-RNA pol II antibody or non-immune IgG and vice versa. The Re-ChIP samples were amplified by PCR using specific primers of the Dspp and Dmp1 genes. Representative results of PCR with the specific primers pairs for Dspp (i) and DMP1 (j) genes. M, DNA markers; Pol II, RNA polymerase II.

Given that GCN5 is among the histone acetyltransferases (HATs) ^31^ and co-expressed with Sp7 in dental cells (Fig. 3bb), both in vitro and in vivo analyses have confirmed the interaction between GCN5 and Sp7 (Fig. 5a–d). Further studies demonstrated that GCN5 acetylates Sp7, a process that can be inhibited by anacardic acid (Fig. 5e, f). To ascertain BMP2 role in Sp7 acetylation, the Sp7 gene was transfected into iMDP3 cells, which were subsequently treated without BMP2, or with BMP2 in combination with trichostatin A. This experiment revealed that BMP2 increased Sp7 acetylation (Fig. 5g). Additionally, GCN5’s activity was dampened by GCN5-shRNA (Fig. 5h), indicating that BMP2-associated GCN5 amplifies Sp7 acetylation and its stability. To determine the mechanisms driving BMP2 influence on Dmp1 and Dspp transcription, Re-ChIP analysis was employed to investigate whether GCN5 interacts with RNA polymerase II (RNA Pol II). RNA Pol II is crucial for initiating gene transcription ^34^. The results showed that GCN5 is recruited to RNA Pol II within the Dmp1 and Dspp promoters (Fig. 5i, j), indicating that BMP2 activity encompasses RNA processing, chromatin remodeling, and regulation of Dmp1 and Dspp expression via GCN5/Sp7.

### GCN5 acetylates histone H3 and governs chromatin remodeling in Dmp1 and Dspp promoters via BMP2

**Figure 6:**
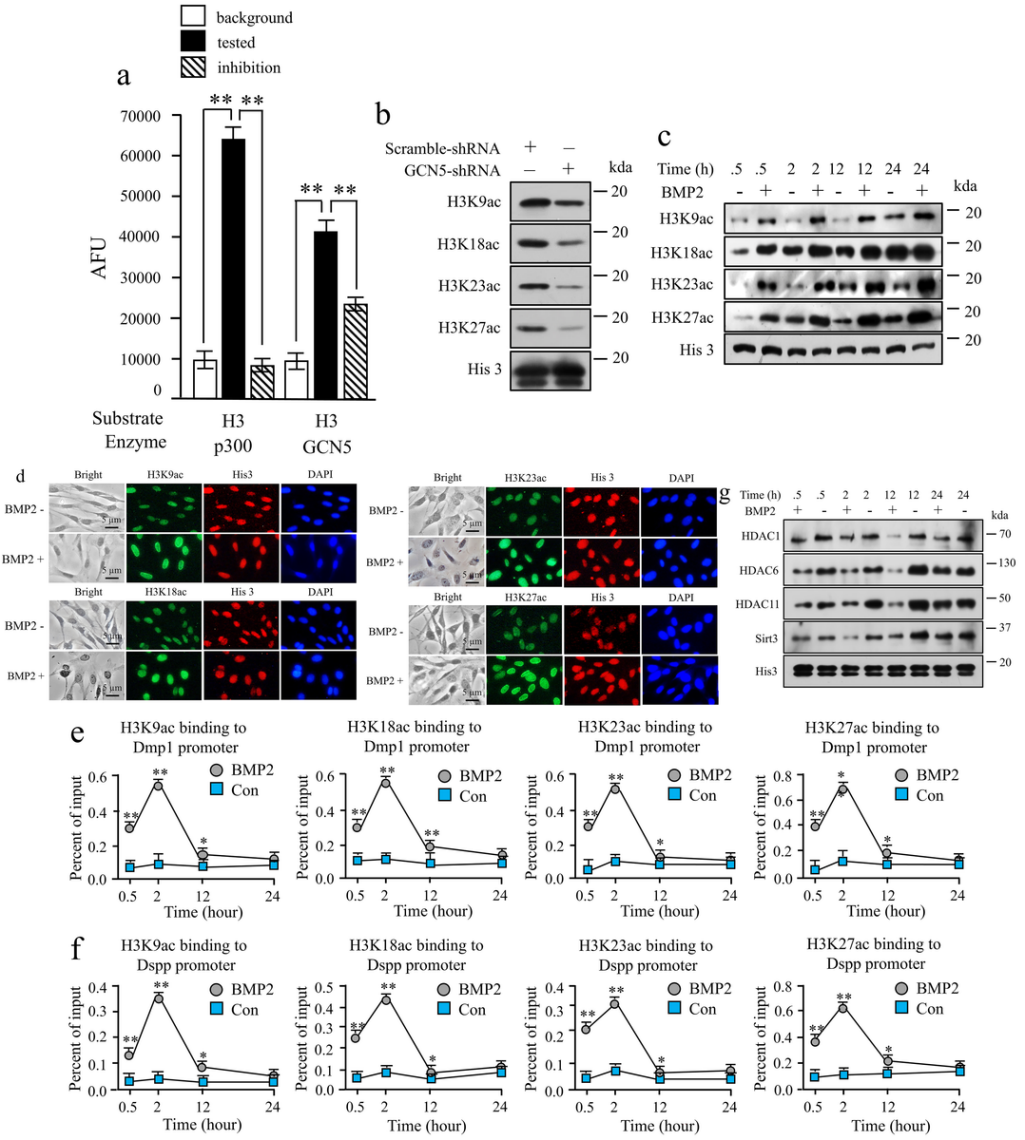
GCN5 acetylates histone H3 and regulates chromatin remodeling in Dmp1 and Dspp promoters by BMP2. (a) p300 and GCN5 acetylated H3, however, p300 and GCN5 activity were inhibited by anacardic acid. p300 and GCN5 (50 ng) activity assays were carried out with the fluorescent HAT assay using either GCN5 (50 ng) or p300 (50 ng), histone 3 peptide substrate (residues 5–23 of human H3, 50 μM), or acetyl-CoA (50 μM) and anacardic acid as an inhibitor (15 μM). AFU, arbitrary fluorescence units. (b). The cells were transfected with either scramble-shRNA or shRNA-GCN5 plasmid for 24h, 48h, and 72h. The expression of His H3 at different lysine residues, and β-actin was detected by Western blotting. (c) The cells were treated with or without 100 ng/ml of recombinant BMP2 for the given periods, and whole proteins were isolated. Expression of histone H3 (His 3), H3K9ac, H3K18ac, H3K23ac and H3K27ac were assayed by Western blotting. (d) Cells were treated with or without 100 ng/ml of recombinant BMP2 for 2h. Immunohistochemistry was performed using His 3, H3K9ac, H3K18ac, H3K23ac, and H3K27ac antibodies. The nucleus was stained by DAPI. BMP2 induces His 3 acetylation at lysine residues and nuclear translocation. (e, f) Dynamic binding of acetylated histone H3 at lysine residues in the Dmp1 and Dspp chromatins mediated by BMP2. Cells were treated with or without BMP2 for the given time points. Dynamic binding activity of H3K9ac, H3K18ac, H3K23ac, and H3K27ac in the Dmp1 (e) and Dspp (f) promoters medicated by BMP2 was analyzed by ChIP assay. Mean data ± S.E. from three independent qPCR experiments in triplicate was plotted. t-test, *p<0.05 and **p<0.01, show significant differences. (g) Effect of BMP2 on the expression of histone deacetylases (HDACs) in iMDP3 cells. The cells were treated with or without 100 ng/ml of recombinant BMP2 for 0.5h, 2h, 12h, and 24h, and whole proteins were isolated. Expression of HDAC1, HDAC6, HDAC11, and Sirt3 was analyzed by Western blot assay. His protein was used for internal control. His3, histone H3.

Through in vitro and in vivo assays, it was demonstrated that GCN5 acetylates histone H3 (H3) (Fig. 6a, b). Inhibitors such as anacardic acid and GCN5-shRNA repressed GCN5-induced H3 acetylation. To explore BMP2 impact on chromatin remodeling, iMDP3 cells were treated with or without recombinant BMP2. Western blotting and immunohistochemistry assays unveiled multiple acetylation sites on H3 that were affected by BMP2. Acetylation was observed at several lysine residues including H3K9ac, H3K18ac, H3K23ac, and H3K27ac (Fig. 6c, d), indicating that BMP2 regulates H3 acetylation through GCN5. Investigating BMP2 effect on chromatin remodeling within Dmp1 and Dspp promoters, iMDP3 cells were treated with or without BMP2 over various time periods. ChIP analysis illustrated that BMP2 dynamically facilitated H3 binding to the Dmp1 and Dspp promoters (Fig. 6e, f). Additionally, BMP2 downregulated the expression of histone deacetylases (HDACs) (Fig. 6g). In light of these results, it’s suggested that BMP2 triggers GCN5 phosphorylation and intracellular translocation, consequently promoting chromatin opening via H3 acetylation and suppressing HDAC activity, thus modulating the balance between HAT and HDAC functions.

## DISCUSSION

BMP2, a member of the TGF-β family, possesses significant roles in embryonic development and tissue formation ^12–14^. Its influence on the proliferation and differentiation of dental mesenchymal progenitor/stem cells has been well documented ^16^. Operating as a ligand, BMP2 initiates multiple signaling processes that regulate cell homeostasis and differentiation. Its functions are intricate, given its involvement in Smad-dependent and Smad-independent pathways. In the former, BMP2 binds to cellular membrane receptors like BMP Receptor Type-I (BMPRI) and BMP Receptor Type-II (BMPRII), activating Smad1/5/8 phosphorylation. This results in the formation of a heteromeric complex with co-Smad (Smad4), translocating into the nucleus to activate downstream gene expression ^12^. The latter pathway involves intracellular phosphorylation of various kinases such as p38 mitogen-activated protein kinase and extracellular signal-regulated protein kinase (Erk), orchestrating diverse downstream signaling events ^12,14^. Global null Bmp2 gene in murine leads to embryonic lethality ^38^. In this study, Bmp2 cKO mice were generated, showing delayed dental mesenchymal cell differentiation and abnormal tooth morphologies resembling DGI ^6,9^ (Fig. 1). DGI is linked to mutations in the DMP1 and DSPP genes ^7,8^.

To decipher the Bmp2 signaling pathway during odontogenesis, RNA-seq and bioinformatics were harnessed to compare gene expression profiles between Bmp2 wild-type and knockout dental mesenchymal cells, revealing diminished Dlx3, Dmp1, Dspp, and Sp7 expressions in Bmp2 null mice (Figs. 2 and 3). Bmp2 mediates the expression of Dmp1 and Dspp genes in dental mesenchymal cells via Dlx3 and Sp7. Dlx3 and Sp7, as transcription factors, are pivotal for odontoblast and osteoblast differentiation. Dlx3 and Sp7 are distinctly expressed in tooth tissues during tooth development and formation ^14,22^. Their mutations in mice and humans correlate with tooth and tissue development anomalies ^14,23,29^. Remarkably, we observed co-expression of Dlx3 and Sp7 within dental mesenchymal cell nuclei, where they interact synergistically to activate gene transcriptions. Bmp2-induced nuclear translocation of Dlx3 and Sp7, along with enhanced binding to Dspp regulatory elements, were identified (Fig. 4). Additionally, Dlx3 positively influenced Sp7 expression via binding to Sp7 promoter sites. However, Sp7 had no reciprocal impact on Dlx3. This indicates that Dlx3 regulates Dmp1 and Dspp transcription through direct or indirect actions, potentially through Sp7 signaling. Furthermore, the study highlights Bmp2 role in inducing nuclear translocation of Dlx3 and Sp7, evidenced by their reduced expression within nuclei in odontoblasts of Bmp2 cKO mice (Figs. 2 and 3). Intriguingly, bioinformatics analyses suggest connections between Bmp2, protein kinases, and Dlx3, Dmp1, Dspp, and Sp7 (Figs. 3 and S3). This study unveils that Bmp2 orchestrates phosphorylation of Akt and Erk^42/44^ kinases, facilitating nuclear translocation of Dlx3 and Sp7 via phosphorylation at specific residues (Tyr and Thr). Dysregulated Bmp2 signaling disrupts odontogenesis, reducing Dlx3 and Sp7 expressions, hindering their nuclear translocation, and subsequently diminishing Dmp1 and Dspp expressions in dental mesenchymal cells. This disruption leads to interrupted odontoblastic cell differentiation and dentin formation in Bmp2 cKO mice.

GCN5, a histone acetyltransferase (HAT) known for acetylating histones and non-histone proteins at lysine residues, plays a pivotal role in chromatin remodeling and gene transcription activation ^32^. This study establishes a novel connection between BMP2 and GCN5 in the context of Dspp and Dmp1 expressions and odontogenesis. Notably, Bmp2 triggers phosphorylation of GCN5 at Tyr and Thr residues through Akt and Erk^42/44^ kinases. This phosphorylation promotes the nuclear translocation of GCN5, followed by its recruitment to RNA Pol II in Dspp and Dmp1 promoters, ultimately leading to chromatin remodeling and activation of Dmp1 and Dspp transcription (Fig. 5). Additionally, Bmp2 fosters nuclear translocation of both GCN5 and Sp7, where GCN5 acetylates Sp7. Bmp2-induced GCN5-driven Sp7 acetylation enhances Sp7 binding to its binding sites in Dspp and Dmp1 promoters. This process is abrogated by GCN5-shRNA. Furthermore, Bmp2 stimulates H3 acetylation, particularly at lysine sites H3K9ac, H3K18ac, H3K23ac, and H3K27ac. Dynamic augmentation of H3 acetylation at these specific residues in Dspp and Dmp1 promoters is instigated by Bmp2. This signifies that Bmp2-triggered GCN5 activity facilitates H3 acetylation in Dspp and Dmp1 promoters, leading to chromatin remodeling and activation of Dmp1 and Dspp gene transcription. It’s worth noting that Bmp2 also downregulates the expression of several histone deacetylases (HDACs), although the precise mechanism remains undisclosed. It was reported that GCN5 directly acetylates a transcriptional factor, early growth response gene 2 (EGR2), which activates its target gene expression including Runx1, promyelocytic leukemia zinc finger protein (PLZF), interleukin (IL)-2 Rb, and T-bet and regulates invariant natural killer T (iNKT) differentiation and development ^39^. Also, GCN5 regulates pathological cardiac hypertrophy in rat cardiomyocytes and mouse models via facilitation of the transforming growth factor β activated kinase 1 (TAK1)/c-Jun N-terminal kinase (JNK)/p38 signaling ^40^.

The balance between histone acetyltransferases (HATs) and histone deacetylases (HDACs) is pivotal for fine-tuning cellular processes, including gene transcription modulation ^34, 41^. Disturbances in this equilibrium can lead to alterations in cell fates. Bmp2 deficiency culminates in dysregulated downstream gene expressions and disruption of the HATs-HDACs balance, resulting in reduced HATs expression and enhanced HDACs activity, ultimately disrupting tooth development. In this intricate interplay, GCN5 emerges as a key player by participating in RNA Pol II recruitment to Dspp and Dmp1 promoters, thus facilitating initial RNA processes through Bmp2 signaling during odontogenesis. Consequently, this study underscores that Bmp2-mediated tooth-related gene expression, odontoblast differentiation, and odontogenesis are orchestrated through the Akt/Erk42/44/Dlx3/GCN5/Sp7 signaling pathways.

In summation, this investigation reveals a novel Bmp2 pathway during odontogenesis. Dysregulated Bmp2 signaling results in the downregulation of Dmp1, Dspp, Dlx3, and Sp7 genes in dental mesenchymal cells, paralleling the dentin deficiency observed in DGI. Bmp2 intricate signaling cascade involves protein phosphorylation of Dlx3 and Sp7 through Akt and Erk^42/44^ kinases, facilitating their nuclear translocation. The interaction between Dlx3 and Sp7 in nuclei is notable, and Bmp2-mediated Dlx3-Sp7 cooperation dynamically upregulates Dspp and Dmp1 transcription in dental mesenchymal cells. Moreover, Bmp2 spurs GCN5 phosphorylation by Akt/Erk^42/44^ kinases, leading to its nuclear translocation and interaction with Sp7 for acetylation, which augments their gene transcription. Bmp2 also elicits H3 acetylation at specific lysine motifs and prompts its recruitment to RNA Pol II, thereby reshaping Dspp and Dmp1 chromatin. Additionally, Bmp2 suppresses HDAC expression, culminating in the activation of Dspp and Dmp1 transcription and the odontogenic process. In essence, Bmp2 regulatory actions encompass both epigenetic and non-epigenetic signaling pathways, decisively influencing odontoblast differentiation and dentin development.

## Materials and methods

### Animals

Experimental protocols followed ARRIVE (Animal Research: Reporting of In Vivo Experiments) guidelines. Animal protocols were reviewed and approved by the Institute Animal Care and Use Committee at the University of Texas Health Science Center in San Antonio (UTHSCSA). Bmp2 floxed allele (Bmp2^fx/fx^) mice was described previously ^42,43^, and cross-bred with the Sp7-Cre mice (Jackson Laboratory, Farmington, CT, USA) to generate Bmp2 conditional KO (cKO) mice. Bmp2 cKO (Bmp2^ko/ko^) and Bmp2 control mice (Bmp2^fx/fx^, Sp7-Cre, wild type, and Bmp ^fx/−^) were employed, and four animals for each group were used for this study.

### Cell culture

Mouse dental immortalized papilla mesenchymal (iMDP3)^44^, Bmp2^fx/fx^, and Bmp2^ko/ko^ mesenchymal cells ^45^ were cultured in αMEM (Invitrogen, San Diego, CA, USA) with 10% bovine serum and penicillin (100 unit/ml) and streptomycin (100 μg/ml), and grown at 37°C. Human fetal kidney (HEK293T) cells were obtained from ATCC (Manassas, VA, USA) and grown in DMEM (Invitrogen) containing 10% bovine serum plus penicillin (100 unit/ml) and streptomycin (100 μg/ml).

### Microscopic analysis of teeth

Animals were anesthetized with Ketamine (Sigma-Aldrich). The morphology of teeth was observed using a stereomicroscope. X-ray radiography was performed to examine bone and tooth changes recorded by a Faxitron radiograph inspection unit (Field X-ray Corporation, Lincolnshire, IL, USA). Digitized images of the incisors and molars were measured and assayed by the AnalySIS software to calculate the size and width of selected components. Morphology of mandibular incisors from 1-, 3-month-old control and Bmp2 cKO mice was observed by Scanning electron microscopy (SEM). The incisor surface was washed in 0.1M sodium cacodylate buffer and fixed in 2.5% (w/v) glutaraldehyde (Sigma-Aldrich) in 0.1M cacodylate buffer for 20 min and then washed in sodium cacodylate buffer and dehydrated in alcohol. The fractured incisor surface was fixed in hexamethyldisilane and sputter-coated with gold. The specimen was observed by SEM at 20 kV (JEOLJSM 6610 LV; JEOL, Inc., Peabody, MA, USA). For Micro-CT, mouse mandibles from the control and Bmp2 cKO mice were scanned using a high-resolution scanner (Scanco Medical AG, Basserdorf, Switzerland) and The Micro-CT-generated DICOM data were analyzed using Micro-view (GE Healthcare, Milwaukee, WI, USA).

### Mechanical property measurements

Mandibular incisors of 1-month-old control and Bmp2 cKO mice were collected and embedded in Acrymount embedding resin (Electron Microscopy Sciences, Hatfield, PA, USA). Mechanical properties of mandibular incisors were measured using a testing machine and shear and compression tests were performed (ReNew Model 1125 Upgrade Package, MTS Systems Corporation, Eden Prairie, MN, USA). Data were analyzed using Image-Pro Plus Software (Media Cybernetics, Inc., Rockville, MD, USA).

### Immunohistochemistry

The postnatal mouse mandibles were dissected, fixed, embedded, and then frontally sectioned at 4 μm. All experimental methods were followed in accordance with relevant guidelines. The slides were incubated with either anti-Dlx3 or anti-Dmp1, anti-Dsp, and anti-Sp7 antibodies, respectively at 4°C overnight. The sections were treated with IgG as negative control (Dako Carpinteria, CA, USA). Subsequently, the slides were incubated with biotinylated secondary antibody (Vector Laboratory Inc., Burlingame, CA, USA), followed by Vectastain Elite ABC reagent (Vector Laboratory Inc.) and stained with DAB and counterstained with Mayer’s hematoxylin. The slides were observed by light microscopy. For double cell fluorescent immunostaining, iMDP3 cells were treated with or without 100 ng/ml of BMP2 (R&D System Inc., Minneapolis, MN, USA), and fixed with 70% ethanol at room temperature. The cells were blocked with 10% donkey serum. After washing, the cells were added with rabbit anti-Dlx3, goat anti-Sp7, and with rabbit anti-GCN5 and goat anti-Sp7 antibodies overnight at 4°C. After washing, the samples were incubated with the secondary antibodies of donkey anti-rabbit Alexa Fluo^®^ 488 green (1:300) and donkey anti-goat Alex Fluo^®^ 568 red (1:300) for 1h. Hoechst was used for nuclear staining. The slides were observed under fluorescent microscopy (Nikon, TE2000–5, JAN).

### Alkaline phosphatase assay and alizarin red S staining

Cells were grown in differentiation medium (DM) with DMEM containing 10% fetal bovine serum, 1% antibiotics, 10 mM sodium β-glycerophosphate, 50 μg/ml ascorbic acid, and 100 nM dexamethasone and control with DMEM supplemented with 10% fetal bovine serum, 1% antibiotics for 0, 7 and 14 days. Then, the cells were fixed and washed in 1xPBS. In situ alkaline phosphatase (ALP) activity was carried out in accordance with the instructions. For cell mineralization, the cells were cultured in DM for 0, 7, and 14 days and fixed in 10% formalin as well as treated with 1% Alizarin red S dye (pH 4.2).

### RNA extraction and quantitative real-time PCR (RT-qPCR)

The postnatal day 2 mandibles and maxillae of the control and Bmp2 cKO mice were homogenized (Bertin Technology, Rockville, MD, USA). Total RNA was isolated using TRIZOL reagent (Qiagen Inc., Valencia, CA, USA), treated with DNase I (Promega), and purified with the RNeasy Mini kit (Qiagen Inc.). RNA concentration was measured with a Bio-analyzer. Standard protocols were used to generate complementary DNA (cDNA). RT-qPCR was performed to quantitate levels of Dlx3, Dmp1, Dspp, Sp7, and cyclophilin A as an internal control using an ABI 7500 (Applied Biosystems, Foster City, CA, USA) and threshold values were calculated using SDS2 software (Applied Biosystems). Primers for RT-qPCR were represented in Table S1. Gene expression levels normalized to cyclophilin A value was calculated by ^ΔΔ^Ct method. The results from 3 separate experiments in triplicate were performed.

### RNA-seq and gene expression analysis

The Bmp2^fx/fx^ and Bmp2^Ko/Ko^ dental mesenchymal cells were harvested, and RNA was extracted using TRIZOL reagent (Qiagen Inc.). The RNA-seq libraries were prepared from total RNAs in accordance with Illumina’s RNA specimen preparation protocol (Illumina Inc., San Diego, CA, USA). RNAs were barcoded, pooled, and sequenced with a HiSeq 2000 system with the 50 bp single read sequencing protocol and with targeted read counts of around 30 million reads per sample. Paired reads to the UCSC mm9 genome build were mapped by a TopHat2 aligner. To quantify gene expression, HTSeq was used to obtain raw read counts per gene and then converted to RPKM in accordance with gene length and total mapped read count per sample. Gene expression levels were measured by Log2-transformed RPKM. Analysis of differential expression and classification of functional annotation were assessed as described earlier ^46^.

### Gene Ontology (GO) and Kyoto Encyclopedia of genes and genomes (KEGG) analysis

GO analysis was used to study the roles of all differentially expressed mRNAs (https://david.ncifcrf.gov/). DAVID-based KEGG analysis was used to determine the significant pathways related to the differentially expressed mRNAs. Fisher’s exact test and the x2 test were used to select the significant GO categories and pathways. The threshold of significance was *p < 0.05 and **p < 0.01.

### Generation of the protein-protein interaction (PPI) network

The differentially expressed genes (DEGs) were imported into a STRING database, and the species was limited to “Mus musculus” to obtain PPI information. The network relationships with high confidence (≥ 0.75) were screened and imported into Cytoscape 3.6.2 to draw a PPI network diagram.

### Western blot analysis

Tissues and cells were lysed with RIPA lysis buffer (Sigma-Aldrich). Protein levels were calculated by the BCA Protein Assay kit (Pierce Biotechnology Inc., Rockford, IL, USA). The same amounts of proteins were subjected to SDS-PAGE gels and Western blotting using specific antibodies as described above.

### Protein phosphorylation

iMDP3 cells were induced either with or without BMP2 (100 ng/ml) for the given times and lysed with RIPA buffer (Sigma-Aldrich). The cell lysate was separated with SDS-PAGE gel electrophoresis and blotted with anti-Akt, anti-pAkt, anti-Erk^42/44^, anti-pErk^42/44^, anti-p38, anti-p-p38, and β-actin antibodies. To assess the phosphorylation of Dlx3, Sp7, and GCN5 induced by BMP2, cells were treated or untreated with BMP2 (100 ng/ml) and lysed. Dlx3, Sp7 and GCN5 proteins were immunoprecipitated with anti-Dlx3, or anti-Sp7, anti-GCN5 antibody, respectively. Immunoprecipitated Dlx3, Sp7, and GCN5 proteins were immunoblotted by anti-pTyr and anti-pThr antibodies. To ascertain if BMP2 induces phosphorylation of Dlx3, Sp7, and GCN5 at Tyr and Thr residues by Akt and Erk^42/44^ kinases, the cells were stimulated or un-stimulated with BMP2 as well as with or without Akt kinase inhibitor, GSK630693 (10–40 μM) or Erk kinase inhibitor, U0126 (10–40 μM, Sigma-Aldrich), respectively. The cells were lysed, and Dlx3, Sp7, and GCN5 were immunoprecipitated by anti-Dlx3, anti-Sp7, and anti-GCN5 antibodies. BMP2-mediated phosphorylation of Akt and Erk^42/44^ kinases inhibited by U0126 and GSK630693 were analyzed by Western blotting using anti-pAkt and anti-pErk^42/44^ antibodies.

### Chromatin immunoprecipitation (ChIP) and Re-ChIP assays

ChIP was preceded in accordance with the ChIP-IT TM kit (Active Motif). Immunoprecipitation was processed with protein G agarose beads and 10 μg of anti-Dlx3 or anti-Sp7 antibody with end-over-end mixing overnight at 4°C. IgG was a negative control. Immunoprecipitated samples were washed, and cross-links reversed. Recovered materials were incubated with proteinase K and the DNA fragments were purified using DNA purification columns (Qiagen Inc.). To assess the dynamic BMP2 effect of binding of Dlx3 and Sp7 to Dspp and Dmp1 promoters, iMDP3 cells were treated or untreated with BMP2. The cell lysate was precipitated with anti-Dlx3 or anti-Sp7 antibody and DNA fragments were purified and amplified by qPCR. Primers for qPCR were shown in Table S2. To determine the effect of Dlx3 on mouse Sp7 promoter, cells were transfected with Dlx3 expression plasmid. After 48h, the ChIP assay was carried out as described above. DNA fragments were purified and amplified by the primers present in Table S3. For Re-ChIP, chromatin complexes from the first ChIP (anti-GCN5 or anti-pol II) were collected and centrifuged. The supernatant was diluted in Re-ChIP buffer. The second IP antibody (either anti-pol II or anti-GCN5) was added, followed by crosslink reversal and DNA purification. The first ChIP and Re-ChIP products were analyzed by PCR that would amplify the 379-bp and 231-bp fragments of Dspp and Dmp1 genes encompassing the GCN5 and pol II binding sites. The primers for PCR were present in Table S4.

### Electrophoretic mobility shift assay (EMSA)

Nuclear proteins from iMDP3 cells were prepared as previously described ^4^. All manipulations were performed at 4°C. Protein levels were measured by the Bradford assay (Bio-Rad Laboratories, Inc., Hercules, CA, USA). For EMSA, the double-stranded DNA probes were labeled with [γ-^32^P] ATP and T4 polynucleotide kinase. EMSA was carried out described earlier ^4^. All oligonucleotides used in EMSA were shown in Table S5.

### Transfection assay

Plasmids of the mouse Dspp promoters were created and described earlier ^4^. In brief, the Dspp elements between nucleotides (nt) −97 and + 54, −318 and + 54, −791 and + 54, −1,243 and + 54, and − 2,644 and + 54 were inserted into pGL-3 basic luciferase plasmid (Promega), to generate plasmids, p97, p318, p791, p1243, and p2644. These reporter plasmids were co-transfected with either pcDNA-Dlx3 or/and pcDNA-Sp7 plasmids, empty pcDNA3.1 plasmid as control with a pRL-TK plasmid (Promega) into iMDP3 cells by Lipofectamine^2000TM^. For mutant DNA constructs, mutant plasmids were created by a mutagenesis kit using p318 DNA construct as a template (Invitrogen). DNA sequencing was employed to verify corrective constructs. After 48h transfection, the cells were collected, and Dspp promoter activity was assayed with the dual luciferase reporter assay system. The Dspp promoter activity was measured by the ratio of firefly/Renilla luciferase for each construct. The experimental data are present to the mean ± S.E. from 3 separate experiments in triplicate.

### Immunoprecipitation

For in vitro protein-protein interaction, full length, NH_2_- and COOH-fragments of mouse Dlx3, Sp7, and GCN5 cDNAs were subcloned into pGEX tagged with glutathione fusion protein gene, respectively. The pGST fusion protein expression and purification were preceded in accordance with the manufacturer’s instruction (Amersham Pharmacia Biotech, Piscataway, NJ, USA). GST-Sp7 proteins were incubated with GST-Dlx3 proteins and with GST-GCN5 proteins, respectively, and then added anti-Sp7 or anti-Dlx3, anti-GCN5 antibodies in the reaction mixtures with end-over-end mixing. IgG was the negative control. After incubation, protein G agarose beads (Invitrogen) were added to the reaction and further incubation. After centrifugation, the supernatant was removed, and an equal volume of 2 x SDS gel-loading buffer was added to the beads and then heated for 5 min. The samples were subjected to Western blotting. For in vivo immunoprecipitation, the full-length, NH_2_- and COOH-domains of Sp7, Dlx3, and GCN5 genes were inserted into mammalian expression vector pcDNA tagged with Flag or c-Myc (Sigma-Aldrich), respectively. iMDP3 and HEK293 cells were transfected with pcDNA-Flag-Sp7, pcDNA-Myc-Dlx3 and pcDNA-Flag-Sp7, pcDNA-Myc-GCN5 genes. After 48h transfection, the cells were washed and scraped. The scarped samples were centrifuged. The supernatant was collected and rotated after adding anti-Flag or anti-Myc antibody (Thermo Fisher Scientific), further incubated with protein G agarose beads (Invitrogen). After the reaction, the samples were washed with lysis buffer and added SDS gel-loading buffer. After centrifugation, the supernatant was collected, electrophoresed to SDS-PAGE gels and a Western blotting assay was performed.

### Sp7 and histone H3 acetylation by GCN5

In vitro Sp7 acetylation was performed by a HAT assay kit (Active Motif). In brief, GST fusion Sp7 (5μg) or 50 μM of histone H3 peptide (Active motif) was incubated with 500 ng of recombinant p300 catalytic domain (Active motif) or recombinant GCN5 protein (Active motif). For acetylation inhibition, anacardic acid (150 μM) was added to the reaction. After incubation, the reaction mixture was added by stop solution, followed by 20 μl of the peptide substrate. 100 μl of the final developing solution was then added to each well and incubated for 15 min in dark at room temperature. The samples were read at fluorescence with excitation at 360–390 nm and emission at 450 to 470 nm on a SpectraMAX Gemini XS plate reader (Molecular Devices, San Jose, CA, USA). To determine the GCN5 effect on Sp7 acetylation in vivo, pcDNA-Sp7, and pcDNA-GCN5 or pcDNA3.1 as control were transfected into iMDP3 cells respectively. After 48h transfection, the cells were washed, lysed, and centrifuged. The supernatant was collected and rotated after adding an anti-Sp7 antibody, then incubated with protein G agarose beads (Invitrogen). After the reaction, the beads were washed with lysis buffer, added SDS gel-loading buffer, and boiled. After centrifugation, the supernatant was collected and electrophoresed SDS-PAGE gel and blotted with anti-Sp7 and anti-acetyl-lysine antibodies (Santa Cruz Biotechnology Inc.). For in vivo assay of Sp7 acetylation induced by BMP2, the pcDNA-Myc-Sp7 vector was transfected into iMDP3 cells and then treated or untreated with BMP2 (100 ng/ml), and with or without 60 nm of Trichostatin A for 2h, 12h, and 24h and then lysed. The co-immunoprecipitation was carried out as described above using an anti-Myc antibody to immuno-precipitate the complexes and subjected to Western blot using anti-Sp7 and anti-acetyl lysine antibodies.

## Statistical analysis

Experimental data are shown as means ± S.D. from 3 independent experiments in triplicate. The difference in statistical significance was analyzed with a one-way t-test using GraphPad Prism8 software (GraphPad Software, Inc. La Jolla, CA, USA). The statistical differences among various groups were significant at *p < 0.05 and **p < 0.01.

## Data Availability

All data associated with this study are presented in the paper.
